# Heterogeneous burden of lung disease in smokers with borderline airflow obstruction

**DOI:** 10.1186/s12931-018-0911-z

**Published:** 2018-11-20

**Authors:** Cheryl S. Pirozzi, Tian Gu, Pedro M. Quibrera, Elizabeth E. Carretta, MeiLan K. Han, Susan Murray, Christopher B. Cooper, Donald P. Tashkin, Eric C. Kleerup, Igor Barjaktarevic, Eric A. Hoffman, Carlos H. Martinez, Stephanie A. Christenson, Nadia N. Hansel, R. Graham Barr, Eugene R. Bleecker, Victor E. Ortega, Fernando J. Martinez, Richard E. Kanner, Robert Paine, for the NHLBI SubPopulations and InteRmediate Outcome Measures In COPD Study (SPIROMICS), Neil E. Alexis, Wayne H. Anderson, R. Graham Barr, Eugene R. Bleecker, Richard C. Boucher, Russell P. Bowler, Elizabeth E. Carretta, Stephanie A. Christenson, Alejandro P. Comellas, Christopher B. Cooper, David J. Couper, Gerard J. Criner, Ronald G. Crystal, Jeffrey L. Curtis, Claire M. Doerschuk, Mark T. Dransfield, Christine M. Freeman, MeiLan Lan K. Han, Nadia N. Hansel, Annette T. Hastie, Eric A. Hoffman, Robert J. Kaner, Richard E. Kanner, Eric C. Kleerup, Jerry A. Krishnan, Lisa M. LaVange, Stephen C. Lazarus, Fernando J. Martinez, Deborah A. Meyers, John D. Newell, Elizabeth C. Oelsner, Wanda K. O’Neal, Robert Paine, Nirupama Putcha, Stephen I. Rennard, Donald P. Tashkin, Mary Beth Scholand, J. Michael Wells, Robert A. Wise, Prescott G. Woodruff, Lisa Postow, Thomas Croxton

**Affiliations:** 1 Division of Pulmonary and Critical Care Medicine, Department of Internal Medicine, University of Utah, 26 N 1900 E, Salt Lake City, UT 84132 USA; 2 Department of Biostatistics, University of Michigan, Ann Arbor, MI USA; 3 Department of Biostatistics, Collaborative Studies Coordinating Center, University of North Carolina at Chapel Hill, Chapel Hill, NC USA; 4 Department of Internal Medicine, University of Michigan, Ann, MI USA; 5 Department of Medicine, UCLA David Geffen School of Medicine, Los Angeles, CA USA; 6 Department of Radiology, University of Iowa, Iowa City, IA USA; 7 Department of Medicine, University of California, San Francisco, CA USA; 8 Department of Medicine, Johns Hopkins University, Baltimore, MD USA; 9 Department of Medicine, Columbia University, New York, NY USA; 10 Department of Medicine, University of Arizona, Tucson, AZ USA; 11 Department of Medicine, Wake Forest University, Winston-Salem, NC USA; 12 Department of Medicine, Weill Cornell Medical College, New York, NY USA; 13 Department of Veterans Affairs Medical Center, Salt Lake City, UT USA

**Keywords:** Chronic obstructive pulmonary disease, Pulmonary function tests, Spirometry, Airway obstruction, Emphysema, Forced expiratory volume, Maximal Midexpiratory flow rate

## Abstract

**Background:**

The identification of smoking-related lung disease in current and former smokers with normal FEV_1_ is complex, leading to debate regarding using a ratio of forced expiratory volume in 1 s to forced vital capacity (FEV_1_/FVC) of less than 0.70 versus the predicted lower limit of normal (LLN) for diagnosis of airflow obstruction. We hypothesized that the discordant group of ever-smokers with FEV_1_/FVC between the LLN and 0.70 is heterogeneous, and aimed to characterize the burden of smoking-related lung disease in this group.

**Methods:**

We compared spirometry, chest CT characteristics, and symptoms between 161 ever-smokers in the discordant group and 940 ever-smokers and 190 never-smokers with normal FEV_1_ and FEV_1_/FVC > 0.70 in the SPIROMICS cohort. We also estimated sensitivity and specificity for diagnosing objective radiographic evidence of chronic obstructive pulmonary disease (COPD) using different FEV_1_/FVC criteria thresholds.

**Results:**

The discordant group had more CT defined emphysema and non-emphysematous gas trapping, lower post-bronchodilator FEV_1_ and FEF_25–75_, and higher respiratory medication use compared with the other two groups. Within the discordant group, 44% had radiographic CT evidence of either emphysema or non-emphysematous gas trapping; an FEV_1_/FVC threshold of 0.70 has greater sensitivity but lower specificity compared with LLN for identifying individuals with CT abnormality.

**Conclusions:**

Ever-smokers with normal FEV_1_ and FEV_1_/FVC <  0.70 but > LLN are a heterogeneous group that includes significant numbers of individuals with and without radiographic evidence of smoking-related lung disease. These findings emphasize the limitations of diagnosing COPD based on spirometric criteria alone.

**Electronic supplementary material:**

The online version of this article (10.1186/s12931-018-0911-z) contains supplementary material, which is available to authorized users.

## Background

Airflow obstruction is a hallmark of chronic obstructive pulmonary disease (COPD), and by current recommendations [1] is confirmed by a reduced ratio of forced expiratory volume in 1 s (FEV_1_) to forced vital capacity (FVC). To simplify the diagnosis of airflow obstruction, a fixed cut-off ratio of FEV_1_/FVC (FEV_1_/FVC <  0.70) is often used instead of predicted lower limit of normal (LLN) (FEV_1_/FVC < LLN), defined as the lower fifth percentile of a reference population. [2, 3]

Because the predicted normal FEV_1_/FVC declines with age, a fixed cut-off ratio of FEV_1_/FVC <  0.70 has the potential for misclassification and over diagnosis in the elderly, [4–10] while using predicted LLN may better predict adverse clinical outcomes [11] and more accurately predict all-cause mortality. [4] Although there is a group of younger individuals for whom LLN is > 0.7, a particular challenge is presented by subjects who fall in a “discordant” group with FEV_1_/FVC ratio > LLN but < 0.7. Compared to subjects with FEV_1_/FVC > 0.70, the individuals in this discordant group have been found to have greater emphysema, airway wall thickening, and gas trapping, as well as greater risk for chronic obstructive pulmonary disease (COPD)-related hospitalization, emergency department visits, and mortality [12–16]. There has been recent interest in characterizing patients with mild smoking-related lung disease as evidenced by symptoms and radiographic abnormalities despite normal spirometry, [17, 18] highlighting the limitations of using spirometric criteria alone for diagnosis of COPD. We hypothesized that the discordant group of ever-smokers with FEV_1_/FVC between the LLN and 0.70 is heterogeneous, containing some individuals with smoking-related lung disease and some with changes in lung function related to aging. We address this hypothesis by characterizing clinical, radiographic and physiologic features of ever-smokers in this discordant group and comparing them with the group of individuals with FEV_1_/FVC > 0.70.^1^

## Methods

### SPIROMICS study methods

SPIROMICS is a multicenter prospective cohort study that has enrolled 2981 participants including never-smokers, smokers without airway obstruction and smokers with mild, moderate and severe COPD, with the goals of identifying new COPD subgroups and intermediate markers of disease progression [19]. Participants were 40–80 years old at baseline. “Smokers” were defined as current or former smokers with lifetime smoking history of greater than 20 pack-years. The study design and exclusion criteria have previously been described [19]. The research protocol was approved by the institutional review boards of all participating institutions and all participants gave written informed consent.

### Subjects and measure of exposure

We analyzed data for three groups of subjects included in SPIROMICS: Group 1) current or former smokers (ever-smokers) with normal post-bronchodilator FEV_1_ and FEV_1_/FVC > LLN but < 0.70 (**discordant group,** *n* = 161); Group 2) ever-smokers with normal FEV_1_ and FEV_1_/FVC > 0.70 (*n* = 940); and Group 3) never-smokers with normal FEV_1_ and FEV_1_/FVC > 0.70 (*n* = 190). In a supplementary analysis we also compared outcomes with a Group 4) patients with FEV_1_/FVC in the 75% quartile of those less than LLN (*n* = 379).

### Pulmonary function methods

Pulmonary function testing was performed and interpreted according to the 2005 ATS/ERS guidelines; post-bronchodilator spirometric measurements were used for analysis [20, 21]. NHANES III spirometric references values were used to calculate percent predicted values and LLN [22].

### Outcomes

We compared respiratory symptoms, quality of life, medication use, CT metrics, FEV_1_% predicted, forced expiratory flow rate between 25 and 75% of FVC or maximum mid-expiratory flow (FEF_25–75_), 6 min walk distance (6MWD), and two prospective variables: annual FEV_1_ change and exacerbation rate, between the three groups. Chronic bronchitis was defined as patient reported cough with sputum for at least 3 months for ≥2 years. Dyspnea was defined by the modified Medical Research Council (mMRC) dyspnea score, [23] stratified into two groups as mMRC ≥2 (moderate or severe dyspnea) vs mMRC 0–1 (mild or no dyspnea). Respiratory symptoms were also measured by the COPD Assessment Test (CAT) [24]. Quality of life was measured by the St. George’s Respiratory Questionnaire (SGRQ) [25]. Medication use was defined as patient-reported regular use of inhaled bronchodilators and/or inhaled steroid. Annual FEV_1_ change was defined using a regression model that incorporated the total number of study visits and spirometry measurements available for each participant. Each participant had a minimum of two spirometry measurements at least 200 days apart, with follow up ranging from 266 to 1749 days. Exacerbation rate was measured as the number of patient-reported events requiring health care utilization in the first year after study enrollment.

Multidetector-row computed tomography (MDCT) scans at full inspiration and full expiration were performed at the SPIROMICS baseline visit. Emphysema was defined using a threshold of <− 950 Hounsfield Units on full inspiration. Airway wall thickening was defined as the square root of the airway wall area for a standardized airway with an internal perimeter of 10 mm (Pi10) [26]. Parametric Response Mapping (PRM) was used to define functional small airways disease (fSAD), a measure of non-emphysematous gas trapping, as the percent of voxels with CT attenuation values > − 950 HU on the inspiratory exam and < − 856 HU on the expiratory scan, as previously described [27] using Imbio Lung Density software (Imbio, Minneapolis, MN).

### Statistical analysis

Comparisons of categorical predictors across groups 1, 2 and 3 used chi-squared tests. For continuous variables, ANOVA was used to test for overall differences between the 3 groups; pairwise comparisons of continuous outcomes between any two groups were based on t-tests. [28] Multivariable linear regression was used to compare continuous measures (emphysema, fSAD, CT metrics, 6MWD, CAT score, quality of life, FEV_1_, and FEF_25–75_) between groups, adjusted for age, sex, race, smoking history (pack-years) and current smoking (yes/no). Multivariable logistic regression was used to compare binary clinical outcomes (emphysema present, fSAD present, chronic bronchitis, mMRC Dyspnea, and medication use) adjusted for the same patient characteristics described above.

Quantile regression models [29] applied to healthy never -smokers estimated the 95th percentile for PRM emphysema and, separately, the 95th percentile for PRM fSAD for a normal patient based on their age, sex, BMI and the scanner used. Hereafter, these estimated 95th percentiles will be used to define the upper limit of normal (ULN) for these PRM measures according to patient/scanner characteristics. Presence of emphysema or fSAD was defined when an individual’s observed PRM emphysema percent or PRM fSAD percent was greater than the estimated ULN for a normal patient with similar patient/scanner characteristics. Sensitivity and specificity of each FEV_1_/FVC cut-off for identifying individuals with radiographic CT evidence of smoking-related lung disease manifest as either emphysema and/or fSAD were estimated.

## Results

We compared the discordant group (Group 1) with ever-smokers with normal spirometry (Group 2) and never-smokers (Group 3). The characteristics of the three groups are shown in Table 1. The discordant group had more male and white participants and was older than the other two groups.

**Table 1:** Baseline characteristics for the three groups

Variable	Group 1 Ever-smokers, Normal FEV_1_, $\frac{\mathrm{FEV}1}{\mathrm{FVC}}$ < 0.7 and > LLN (Discordant Group) (*n* = 161)	Group 2 Ever-smokers, Normal FEV_1_, $\frac{\mathrm{FEV}1}{\mathrm{FVC}}$> 0.7 (*n* = 940)	Group 3 Never-smokers, Normal FEV_1_, $\frac{\mathrm{FEV}1}{\mathrm{FVC}}$> 0.7 (*n* = 190)	*P*-value
Sex (% male)	70.8%	49.0%	37.9%	< 0.001*
Race (% white)	89.4%	68.2%	70.7%	< 0.001*
Current smoker (%)	32.5%	50.0%	0%	< 0.001*
Age (mean ± SD)	69.3 ± 6.4	60.4 ± 9.7	56.6 ± 10.2	< 0.001^†^
Smoking history in pack-years (mean ± SD)	48.3 ± 22.2	43.1 ± 27.3	Not Applicable	0.0^‡^

*Chi-Square test

^†^ANOVA

^‡^t-test

**Fig. 1:**
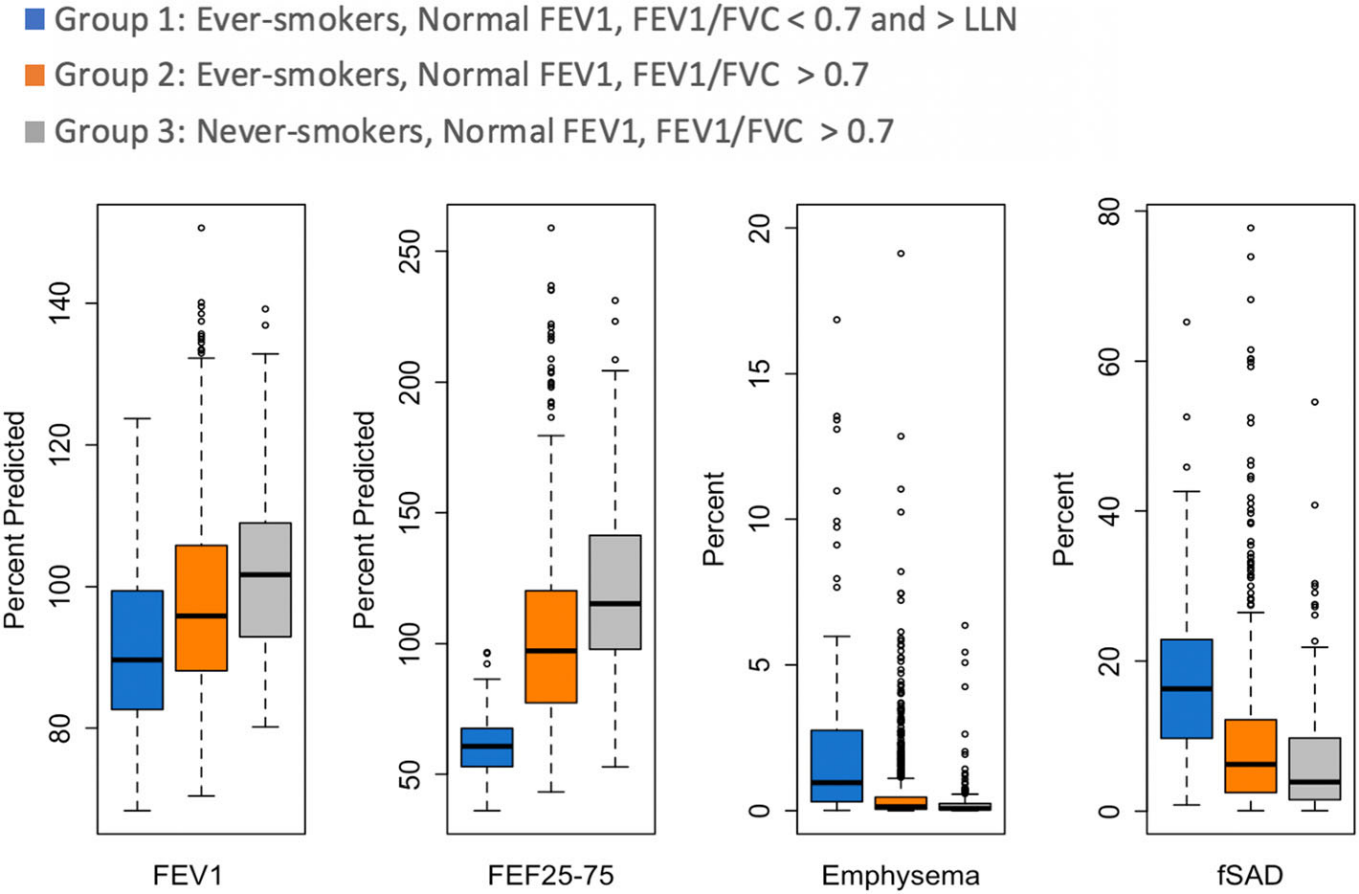
Box plots demonstrating percent of predicted forced expiratory volume in 1 s (FEV_1_%), forced expiratory flow rate between 25 and 75% of forced vital capacity (FEF_25–75%_), percent emphysema, and functional small airways disease by parametric response mapping (fSAD) in the three groups

Compared with ever-smokers with FEV_1_/FVC > 0.70 (Group 2), the discordant individuals (Group 1) had lower post-bronchodilator FEV_1_% predicted (92.1% vs 97.5%, *p* <  0.001) and reduced FEF_25–75_% predicted (61.2% vs 102.3%, p <  0.001) (Table 2, Fig. 1). The two groups of ever-smokers did not differ significantly in 6MWD (437.5 vs 437.2 m, *p* = 0.97), SGRQ (22.5 vs 24.2, *p* = 0.28), or CAT score (10.7 vs 11.3, *p* = 0.36). More smokers in the discordant group reported regular use of either inhaled corticosteroids and/or bronchodilators than either ever-smokers with FEV_1_/FVC > 0.70, Group 2, (34.4% vs. 25.1%, *p* = 0.01) or never-smokers, Group 3 (3.9%, *p* <  0.001). Groups 1 and 2 did not differ in the reported incidence of chronic bronchitis or moderate or severe dyspnea indicated by mMRC score ≥ 2. Nor did they differ with respect to FEV_1_ decline per year, or exacerbations per year (Table 2).

**Table 2:** Comparison of physiologic and clinical variables between ever-smokers with normal FEV_1_ and FEV_1_/FVC > LLN but < 0.70 (“discordant” group), ever-smokers with normal FEV_1_ and FEV_1_/FVC > 0.70, and never-smokers with normal FEV_1_ and FEV_1_/FVC > 0.70

Clinical Outcome	Group 1 Ever-smokers, Normal FEV_1_,$\frac{\mathrm{FEV}1}{\mathrm{FVC}}$ < 0.7 and > LLN (Discordant Group) (*n* = 161)	Group 2 Ever-smokers, Normal FEV_1_, $\frac{\mathrm{FEV}1}{\mathrm{FVC}}$> 0.7 (*n* = 940)	Group 3 Never-smokers, Normal FEV_1_, $\frac{\mathrm{FEV}1}{\mathrm{FVC}}$> 0.7 (*n* = 190)	Overall *p*-value* Unadjusted (Adjusted)	*P*-values for pairwise comparisons (Unadjusted)		
					Group 1 vs. 2	Group 1 vs. 3	Group 2 vs. 3
FEV_1_% predicted	92.1 ± 12.0	97.5 ± 12.8	102.0 ± 11.5	< 0.001 (< 0.001)	< 0.001	< 0.001	< 0.001**
FEF_25–75%_ % predicted	61.2 ± 11.0	102.3 ± 33.4	121.3 ± 32.5	< 0.001 (< 0.001)	< 0.001	< 0.001	< 0.001**
6MWD (m)	437.5 ± 109.6	437.2 ± 97.7	479.3 ± 103.4	< 0.001 (0.49)	0.97	< 0.001	< 0.001**
St George’s Respiratory Questionnaire Total Score	22.5 ± 17.4	24.2 ± 19.1	8.8 ± 10.0	< 0.001 (< 0.001)	0.28	< 0.001	< 0.001**
COPD Assessment Test (CAT)	10.7 ± 7.4	11.3 ± 8.1	4.7 ± 6.0	< 0.001 (< 0.001)	0.36	< 0.001	< 0.001**
Use of either inhaled corticosteroid or bronchodilator	34.4%	25.1%	3.9%	< 0.001 (< 0.001)	0.01	< 0.001	< 0.001†
Chronic bronchitis	17.3%	17.8%	2.1%	< 0.001 (< 0.001)	0.88	< 0.001	< 0.001†
mMRC Dyspnea score ≥ 2	13.8%	13.6%	2.7%	< 0.001 (0.007)	0.95	< 0.001	< 0.001†
Change in FEV_1_ (ml/year)	− 60.5 ± 120.5	−55.2 ± 127.5	−41.2 ± 99.7	0.32 (0.94)	0.64	0.17	0.19**
Exacerbation (#/year)	0.1 ± 0.4	0.1 ± 0.6	0.02 ± 0.1	0.02 (0.21)	0.50	0.13	0.006**

Emphysema = % of voxels with CT attenuation <− 950 Hounsfield Units (HU) on full inspiration. Functional small airways disease = % of voxels with CT attenuation > − 950 HU on the inspiratory exam and < − 856 HU on the expiratory scan, as determined via dynamic image registration (Parametric Response Mapping, PRM). Airway thickening = square root of the wall area for a standardized airway with an internal perimeter of 10 mm (Pi10)

*From likelihood ratio test comparing means of 3 groups from multivariable model with outcomes (rows) and group status as predictors adjusted for age, sex, race, smoking history (pack-years) and current smoking

**p-values from 2 sample t-test

^†^Pairwise *p*-value form Wald test comparing means of 2 groups

The discordant group had a modest but significantly greater percentage of lung with CT scan-defined emphysema than Group 2 (2.1% vs 0.7%, p <  0.001) or Group 3 (0.3%, p <  0.001). Individuals in the discordant group also had significantly increased PRM fSAD as compared to Groups 2 and 3 (18.0% vs. 9.1% and 7.1%, respectively, p <  0.001), without detectable differences in airway thickness (Table 3, Fig. 1). Density plots illustrating the distribution of emphysema and small airways disease are presented in supplementary material (Additional file 1: Figure S1 and Additional file 2: Figure S2).

**Table 3:** Comparison of CT variables between ever-smokers with normal FEV_1_ and FEV_1_/FVC > LLN but < 0.70 (“discordant” group), ever-smokers with normal FEV_1_ and FEV_1_/FVC > 0.70, and never-smokers with normal FEV_1_ and FEV_1_/FVC > 0.70

Variable	Group 1 Ever-smokers, Normal FEV_1_, $\frac{\mathrm{FEV}1}{\mathrm{FVC}}$ < 0.7 and > LLN (Discordant Group) (*n* = 161)	Group 2 Ever-smokers, Normal FEV_1_, $\frac{\mathrm{FEV}1}{\mathrm{FVC}}$> 0.7 (*n* = 940)	Group 3 Never-smokers, Normal FEV_1_, $\frac{\mathrm{FEV}1}{\mathrm{FVC}}$> 0.7 (*n* = 190)	Overall *p*-value* from likelihood ratio test comparing association with group status Unadjusted (Adjusted)	P-values for pairwise comparisons (Unadjusted)		
					Group 1 vs. 2	Group 1 vs. 3	Group 2 vs. 3
Emphysema (%)	2.1 ± 2.9	0.7 ± 2.6	0.3 ± 0.9	< 0.001 (< 0.001)	< 0.001	< 0.001	< 0.001**
Functional small airways disease (%)	18.0 ± 10.6	9.1 ± 10.0	7.1 ± 8.3	< 0.001 (< 0.001)	< 0.001	< 0.001	< 0.001**
Airway wall thickening (Pi10)	3.70 ± 0.01	3.71 ± 0.00	3.69 ± 0.01	< 0.001 (0.17)	0.41	0.01	< 0.001**
Emphysema present > ULN	38.7%	17.4%	8.2%	< 0.001 (< 0.001)	< 0.001	< 0.001	0.004†
CT-defined functional small airway abnormality (fSAD) present > ULN	15.3%	7.8%	2.9%	< 0.001 (0.03)	0.003	< 0.001	0.03†
Either emphysema or fSAD present	44%	20.7%	9.4%	< 0.001 (< 0.001)	< 0.001	< 0.001	< 0.001†
Both emphysema and fSAD present	10%	4.5%	1.8%	0.002 (0.23)	0.007	0.005	0.11†

Presence of emphysema = ≥ upper limit of normal (ULN); Presence of fSAD = ≥ upper limit of normal (ULN). Emphysema = % of voxels with CT attenuation <− 950 Hounsfield Units (HU) on full inspiration. Functional small airways disease = % of voxels with CT attenuation > − 950 HU on the inspiratory exam and < − 856 HU on the expiratory scan, as determined via dynamic image registration (Parametric Response Mapping, PRM)

*From likelihood ratio test comparing means of 3 groups from multivariable model with outcomes (rows) and group status as predictors adjusted for age, sex, race, smoking history (pack-years) and current smoking

**P-value from 2 sample t test

^†^Pairwise *p*-value form Wald test comparing means of 2 groups

**Fig. 2:**
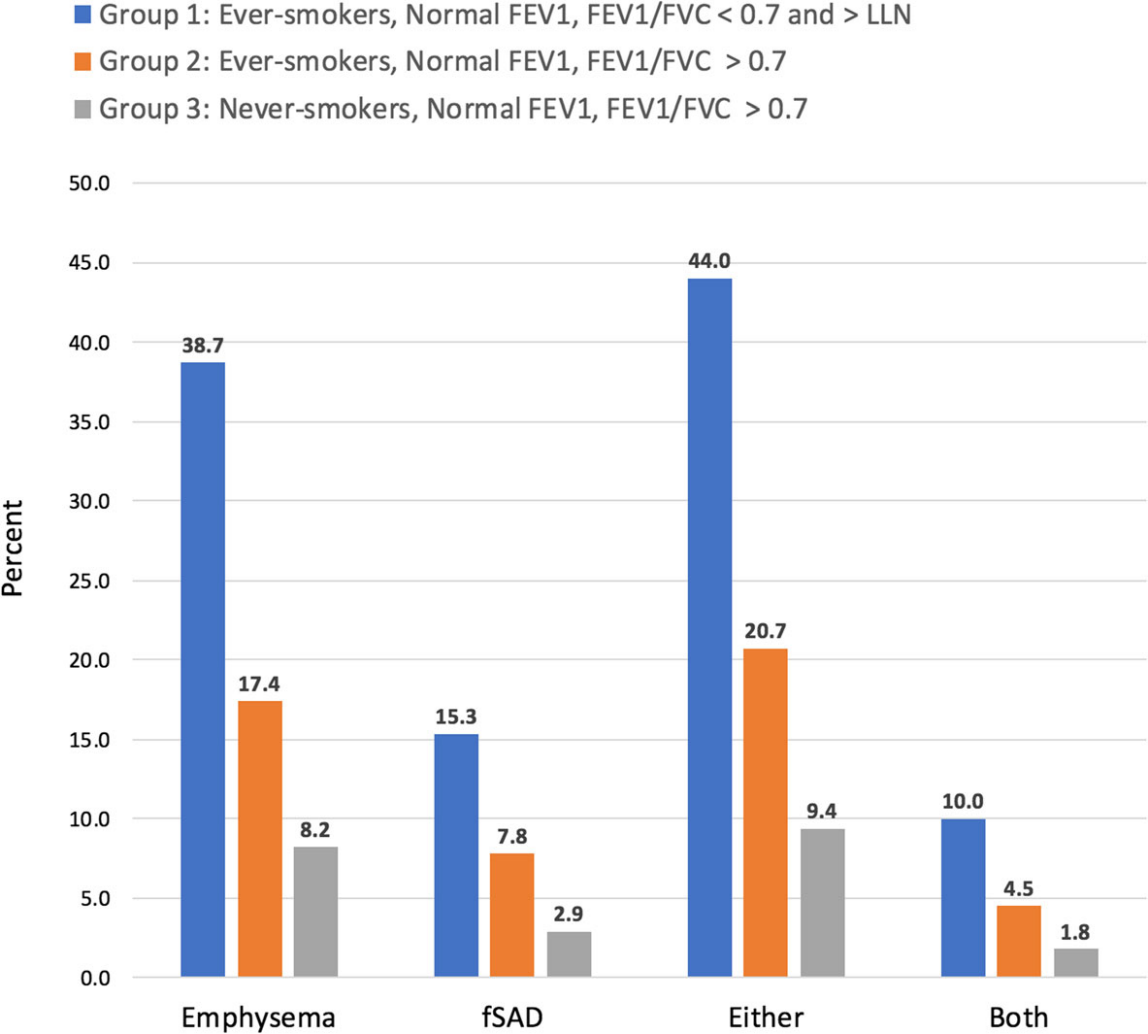
Percent of patients in each group with emphysema and functional small airways disease (fSAD) present greater than the age-adjusted upper limit of normal (ULN) as measured by parametric response mapping (PRM) on chest CT

Using age-adjusted ULN for % emphysema, more individuals in the discordant group met CT criteria for the presence of emphysema compared with Groups 2 and 3 (38.7% vs. 17.4% (p <  0.001) and 8.2% (p <  0.001), respectively). Similarly, using age-adjusted ULN for PRM fSAD, more individuals in the discordant group also met CT criteria for the presence of fSAD compared with Groups 2 and 3 (15.3% vs. 7.8% (*p* = 0.003) and 2.9% (p <  0.001), respectively). In the discordant group, 44% of subjects had CT evidence of smoking-related lung disease, manifest as either emphysema or fSAD, compared with 20.7% (p <  0.001) of Group 2 and 9.4% (p <  0.001) of Group 3 subjects. Conversely, 56% of individuals in the discordant group had CT scans without radiographic evidence of smoking-related lung disease (Table 3, Fig. 2 , Additional file 3: Figure S3).

Within the discordant group, those with CT evidence of smoking-related lung disease did not have significantly greater respiratory symptoms, FEV_1_ decline, exacerbations, or lower FEF_25–75_ compared with those without emphysema or fSAD (Table 4).

**Table 4:** Comparison of prospective FEV_1_ decline, exacerbation rate and respiratory symptoms between those in the discordant group (Ever-smokers with normal FEV_1_, FEV_1_/FVC < 0.70 and > LLN) with CT findings of emphysema or functional small airways disease, and those without

	Ever-smokers with normal FEV_1_, FEV_1_/FVC < 0.70 and > LLN (discordant group, *n* = 161)			
	With fSAD or emphysema (44%)	Without fSAD or emphysema (56%)	*Adjusted *p*-value	Unadjusted *p*-value
Annual change in FEV_1_ (ml/year)	−65.6 ± 113.0	−59.8 ± 130.6	0.26	0.79
Exacerbation (#/year)	0.13 ± 0.42	0.06 ± 0.24	0.90	0.25
COPD Assessment Test (CAT)	11.43 ± 7.44	9.88 ± 7.25	0.55	0.22
Chronic bronchitis (%)	14.3 ± 35.3	18.3 ± 38.9	0.58	0.52
FEF_25–75%_ % predicted	60.0 ± 11.6	62.2 ± 10.7	0.87	0.24

*Multivariate model adjusting for age, gender, race, pack year, and current smoking status

When compared to a fourth group of smokers with FEV_1_/FVC in the 75% quartile of those less than LLN, the discordant group had higher FEV_1_ and FEF_25–75%_, fewer respiratory symptoms and exacerbations, less airway wall thickness and fewer % of people with fSAD, however did not differ in the amount of emphysema or FEV_1_ decline (Additional file 4: Tables S1, S2 and S3).

A history of smoking had a significant association with symptoms in groups defined by either FEV_1_/FVC threshold compared to never-smokers. When compared with never-smokers without airflow obstruction, both groups of ever-smokers had more chronic bronchitis, dyspnea, respiratory symptoms as measured by CAT, and lower quality of life by SGRQ (Table 2).

Finally, we evaluated the sensitivity and specificity of the two thresholds for FEV_1_/FVC for identification of individuals with radiographic evidence of smoking-related lung disease (emphysema > age-adjusted ULN and/or PRM fSAD > age-adjusted ULN). A threshold of FEV_1_/FVC <  0.7 had a sensitivity of 0.85 and specificity of 0.72 for identifying any radiographic abnormality**.** The FEV_1_/FVC < LLN threshold had lower calculated sensitivity **(**0.78) and higher specificity (0.81) (Table 5). Thus the absolute ratio is more sensitive, while the LLN is more specific.

**Table 5:** Calculated sensitivity and specificity for diagnosis of COPD defined by presence of radiographic CT evidence of smoking related lung disease, with emphysema > age adjusted upper limit of normal and/or functional small airways disease > ULN. *N* = 2972. LLN = lower limit of normal

Diagnostic criteria	Sensitivity	Specificity
FEV_1_/FVC < 0.7	0.85	0.72
FEV_1_/FVC < LLN	0.78	0.81

## Discussion

In the SPIROMICS cohort, current or former smokers with normal FEV_1_ who are diagnosed with COPD based on GOLD spirometric criteria, but who do not have airflow obstruction based on the LLN threshold, have more emphysema and functional small airways disease by CT, increased use of inhaled medications, and lower mid-expiratory flow compared with current or former smokers without airway obstruction, defined by FEV_1_/FVC > 0.70. Almost half of individuals in this discordant group have CT evidence of smoking-related lung disease. Nevertheless, the discordant group did not have increased respiratory symptoms (chronic bronchitis, dyspnea, or CAT) or decreased exercise tolerance when compared with individuals with FEV_1_/FVC ratio > 0.7.

We have focused this analysis on individuals in this discordant group for three reasons. First, in reference populations the ratio of FEV_1_/FVC decreases with advancing age, suggesting that use of a fixed threshold of 0.7 may inappropriately classify some individuals. Second, studies of this population may help elucidate the boundaries of normal aging in the setting of cigarette smoking. Finally, there has been recent interest in the clinical picture of smokers who may have smoking-related lung disease in the setting of little or no airflow obstruction [17, 18]. This study contributes to the discussion in each of these three areas.

Our findings support the presence of early/mild disease among individuals in this discordant group and thus provide potential pathophysiologic explanation for previous studies demonstrating increased risk for COPD-related health effects in this group, including increased adjusted risk of death, COPD-related emergency department visits and hospitalizations [13, 15]. These studies suggest that the LLN threshold lacks sensitivity, failing to identify a number of individuals with clinically significant disease.

However, because the predicted FEV_1_/FVC may decline with normal age, using a fixed cut-off ratio of FEV_1_/FVC <  0.70 increases diagnosis of obstruction in the elderly, and in very old adults has the potential to classify changes associated with aging as COPD [4–9]. In a cohort of adults 80 years and older, airflow obstruction defined by FEV_1_/FVC < LLN, but not FEV_1_/FVC between LLN and 0.70, was associated with increased mortality [4]. Similarly, small amounts of emphysema may occur due to aging-related changes rather than as a consequence of early smoking-related disease. In the multiethnic MESA cohort, full-lung CT scans of healthy nonsmokers revealed a small percent of emphysema (median 1.1%) that was increased in men and with age. [30] CT-defined functional small airway abnormality also increases with age [31]. Therefore, the predicted “normal” amount of emphysema and small airways disease increases with aging even in the absence of smoking exposure. An important question is how to distinguish between early/mild COPD and normal aging. In our study we used data from normal individuals to create age-adjusted upper limits of normal for both emphysema and PRM fSAD, suggesting that the CT abnormality we have identified in the discordant group is beyond that associated with normal aging. Our study extends previous findings by including innovative imaging parameters of small airways disease and comparisons with normal lung density [16].

We found significantly reduced FEF_25–75%_ and CT scan evidence of non-emphysematous air trapping in the discordant group. Reduction in mid-expiratory flow is generally assumed to be an indication of small airways disease [32–34]. We did not identify differences in airway wall thickness, manifest as path specific Pi10, associated with our discordant group. However, changes in lumen dimension may mask changes in wall thickening/thinning by this parameter [35]. CT air trapping is also thought to reflect small airways disease and has been associated with lower lung function and accelerated lung function decline [36, 37]. The functional small airways disease measurement using PRM helps to distinguish non-emphysematous air trapping from emphysema on CT [27]. Thus physiologic and CT scan data both point to subtle but potentially clinically important small airways abnormalities in this discordant group. Several studies have suggested that in the natural history of COPD, small airways may become narrowed or lost prior to the onset of emphysema [34, 38–40] and thus these abnormalities may be an earlier indication of smoking-related COPD. We evaluated two prospective variables: exacerbations in the first year after enrollment, and FEV_1_ decline over a period of up to 4 years. Though we did not detect more FEV_1_ decline or exacerbations in the discordant group or those with radiographic emphysema or fSAD, exacerbation rate was overall low in these patients with mild smoking-related lung disease. Longer follow up time will enhance our understanding of the significance of these mild radiographic and physiologic abnormalities as predictors of progression to COPD.

The choice of a threshold of FEV_1_/FVC for diagnosing airflow obstruction may depend on the goals of testing and whether a more sensitive or specific test is preferred. In the SPIROMICS cohort, using a FEV_1_/FVC threshold of 0.70 is more sensitive but less specific for identifying individuals with radiographic manifestations of COPD, while using LLN is more specific but less sensitive. As a screening test for early/mild disease in ever-smokers, a more sensitive test may be preferred. However, identifying airflow obstruction using either FEV_1_/FVC threshold will incorrectly classify individuals.

There are several important features of this study. SPIROMICS is a large multi-center cohort whose subjects are extensively characterized for symptoms, physiology and radiology. MDCT scans performed at baseline allowed detailed assessment of emphysema, air trapping, and airway wall thickness and image analysis by PRM allowed differentiation of non-emphysematous air trapping from emphysema on CT. We recognize several limitations to our study. FEF_25–75%_ is an effort-dependent measurement like FEV_1_ and we cannot exclude a confounding effect of limited effort or frailty. However, the subjects described in this report all had studies that met ATS criteria and had normal FVC. The specificity statistic is biased because the analysis data set is not a random sample. Additionally, never-smokers with FEV_1_/FVC <  0.7 were not included in SPIROMICS and thus could not be compared in this analysis.

## Conclusions

Ever-smokers who have normal FEV_1_ and FEV_1_/FVC <  0.70 but > LLN (discordant group) have on average more emphysema and small airways disease, and increased respiratory medication use compared with those with FEV_1_/FVC > 0.70. This is a heterogeneous group that includes a large number of individuals with CT evidence of either emphysema or non-emphysematous gas trapping, as well as many individuals without radiographic evidence of early smoking-related lung disease for whom it is likely that normal aging accounts for the apparent spirometric abnormality. The diagnosis of early/mild COPD requires a more sophisticated approach that goes beyond currently accepted spirometric criteria.

## Additional files

Additional file 1: **Table S1.** Baseline characteristics for the four groups. **Table S2.** Comparison of physiologic and clinical variables between ever-smokers with normal FEV_1_ and FEV_1_/FVC > LLN but < 0.70 (“discordant” group, Group 1), ever-smokers with normal FEV_1_ and FEV_1_/FVC > 0.70 (Group 2), never-smokers with normal FEV_1_ and FEV_1_/FVC > 0.70 (Group 3) and ever-smokers with FEV_1_/FVC ≤ LLN and > 75th quartile (Group 4). **Table S3.** Comparison of CT variables between ever-smokers with normal FEV_1_ and FEV_1_/FVC > LLN but < 0.70 (“discordant” group, Group 1), ever-smokers with normal FEV_1_ and FEV_1_/FVC > 0.70 (Group 2), never-smokers with normal FEV_1_ and FEV_1_/FVC > 0.70 (Group 3) and ever-smokers with FEV_1_/FVC ≤ LLN and > 75th quartile (Group 4). (DOCX 100 kb)

Additional file 2: **Figure S1.** Density plot of the distribution of emphysema. (PNG 58 kb)

Additional file 3: **Figure S2.** Density plot of the distribution of functional small airways disease. (PNG 55 kb)

Additional file 4: **Figure S3.** Venn diagram illustrating patients within the discordant group (ever-smokers with normal FEV_1_ and FEV_1_/FVC > LLN but < 0.70) who have emphysema, functional small airways disease (fSAD), and both on chest CT imaging. (PDF 34 kb)

Additional file 5: Heterogeneous Burden of Lung Disease in Smokers with Borderline Airflow Obstruction. (DOCX 73 kb)

## Abbreviations

6MWD: 6 min walk distance
CAT: COPD Assessment Test
COPD: Chronic obstructive pulmonary disease
CT: Computed tomography
FEF_25–75_: Forced expiratory flow rate between 25 and 75% of FVC
FEV_1_: Forced expiratory volume in 1 s
fSAD: Functional small airways disease
FVC: Forced vital capacity
LLN: Lower limit of normal
mMRC: Modified Medical Research Council
Pi10: Square root of the airway wall area for a standardized airway with an internal perimeter of 10 mm
PRM: Parametric Response Mapping
SGRQ: St. George’s Respiratory Questionnaire
ULN: Upper limit of normal

## Footnotes

1 Some of the results of these studies have been previously reported in the form of an abstract [41, 42].
