# Supplementary material for: Heterogeneous burden of lung disease in smokers with borderline airflow obstruction
Source: Respir Res. 2018 Nov 20;19:223. doi: 10.1186/s12931-018-0911-z (PMC6245799; doi:10.1186/s12931-018-0911-z)
Supplement: Supplementary file 1 — Table S1. Baseline characteristics for the four groups. Table S2. Comparison of physiologic and clinical variables between ever-smokers with normal FEV1 and FEV1/FVC > LLN but < 0.70 (“discordant” group, Group 1), ever-smokers with normal FEV1 and FEV1/FVC > 0.70 (Group 2), never-smokers with normal FEV1 and FEV1/FVC > 0.70 (Group 3) and ever-smokers with FEV1/FVC ≤ LLN and > 75th quartile (Group 4). Table S3. Comparison of CT variables between ever-smokers with normal FEV1 and FEV1/FVC > LLN but < 0.70 (“discordant” group, Group 1), ever-smokers with normal FEV1 and FEV1/FVC > 0.70 (Group 2), never-smokers with normal FEV1 and FEV1/FVC > 0.70 (Group 3) and ever-smokers with FEV1/FVC ≤ LLN and > 75th quartile (Group 4). (DOCX 100 kb) [file 12931_2018_911_MOESM1_ESM.docx]

**Table S1**: Baseline characteristics for the four groups.

| Variable | **Group 1**  Ever-smokers, Normal FEV_1_,  $\frac{FEV1}{\mathrm{FVC}}<0.7$ and > LLN  (**Discordant Group**)  (n = 161) | **Group 2**  Ever-smokers, Normal FEV_1_,  $\frac{FEV1}{\mathrm{FVC}}$> 0.7  (n = 940) | **Group 3**  Never-smokers, Normal FEV_1_,  $\frac{FEV1}{\mathrm{FVC}}$> 0.7  (n = 190) | **Group 4**  Ever-smokers,  75% quartile < $\frac{FEV1}{\mathrm{FVC}}\leq LLN$  (n = 379) | P-value |
| --- | --- | --- | --- | --- | --- |
| Sex (% male) | 70.8% | 49.0% | 37.9% | 46.7% | <0.001^*^ |
| Race (% white) | 89.4% | 68.2% | 70.7% | 75.5% | <0.001^*^ |
| Current smoker (%) | 32.5% | 50.0% | 0% | 49.3% | <0.001^*^ |
| Age (mean ± SD) | 69.3 ± 6.4 | 60.4 ± 9.7 | 56.6 ± 10.2 | 62.1 ± 8.7 | <0.001^†^ |
| Smoking history in pack-years (mean ± SD) | 48.3 ± 22.2 | 43.1 ± 27.3 | Not Applicable | 50.0 ± 23.9 | <0.001^†^ |

^*^Chi-Square test

^†^ANOVA

**Table S2**

Comparison of physiologic and clinical variables between ever-smokers with normal FEV_1_ and FEV_1_/FVC > LLN but < 0.70 (“discordant” group, Group 1), ever-smokers with normal FEV_1_ and FEV_1_/FVC > 0.70 (Group 2), never-smokers with normal FEV_1_ and FEV_1_/FVC > 0.70 (Group 3) and ever-smokers with FEV_1_/FVC ≤ LLN and > 75^th^ quartile (Group 4).

| Clinical Outcome | **Group 1**  Ever-smokers, Normal FEV_1_,  $\frac{FEV1}{\mathrm{FVC}}<0.7$ and > LLN (**Discordant Group**)  (n = 161) | **Group 2**  Ever-smokers, Normal FEV_1_,  $\frac{FEV1}{\mathrm{FVC}}$> 0.7  (n = 940) | **Group 3**  Never-smokers, Normal FEV_1_,  $\frac{FEV1}{\mathrm{FVC}}$> 0.7  (n = 190) | **Group 4**  Ever-smokers,  75% quartile < $\frac{FEV1}{\mathrm{FVC}}\leq LLN$  (n = 379) | Overall  p-value*  Unadjusted  (Adjusted) | P-values for pairwise comparisons (Unadjusted) | | | | | |
| --- | --- | --- | --- | --- | --- | --- | --- | --- | --- | --- | --- |
|  |  |  |  |  |  | Group  1 vs. 2 | Group  1 vs. 3 | Group  1 vs. 4 | Group  2 vs. 3 | Group  2 vs. 4 | Group  3 vs. 4 |
| FEV_1_ % predicted | 92.1 ± 12.0 | 97.5 ± 12.8 | 102.0 ± 11.5 | 76.9 ± 16.2 | < 0.001  (< 0.001) | < 0.001 | < 0.001 | < 0.001 | < 0.001 | < 0.001 | < 0.001** |
| FEF_25-75%_ % predicted | 61.2 ± 11.0 | 102.3 ± 33.4 | 121.3 ± 32.5 | 45.3 ± 11.9 | < 0.001  (< 0.001) | < 0.001 | < 0.001 | < 0.001 | < 0.001 | < 0.001 | < 0.001** |
| 6MWD (m) | 437.5± 109.6 | 437.2 ± 97.7 | 479.3 ± 103.4 | 422.2 ± 128.1 | < 0.001 (0.17) | 0.97 | < 0.001 | 0.13 | < 0.001 | 0.02 | < 0.001** |
| St George’s Respiratory Questionnaire Total Score | 22.5 ± 17.4 | 24.2 ± 19.1 | 8.8 ± 10.0 | 34.6 ± 20.0 | < 0.001 (<0.001) | 0.28 | < 0.001 | < 0.001 | < 0.001 | < 0.001 | < 0.001** |
| COPD Assessment Test (CAT) | 10.7 ± 7.4 | 11.3 ± 8.1 | 4.7 ± 6.0 | 14.5 ± 7.7 | < 0.001 (<0.001) | 0.36 | < 0.001 | < 0.001 | < 0.001 | < 0.001 | < 0.001** |
| Use of either inhaled corticosteroid or bronchodilator | 34.4% | 25.1% | 3.9% | 34.6% | < 0.001  (< 0.001) | 0.01 | <0.001 | 0.96 | <0.001 | 0.003 | <0.001** |
| Chronic bronchitis | 17.3% | 17.8% | 2.1% | 24.3% | < 0.001 (<0.001) | 0.88 | <0.001 | 0.05 | <0.001 | <0.001 | <0.001^†^ |
| mMRC Dyspnea score ≥ 2 | 13.8% | 13.6% | 2.7% | 21.6% | < 0.001 (<0.001) | 0.95 | <0.001 | 0.03 | <0.001 | <0.001 | <0.001^†^ |
| Change in FEV_1_ (ml/year) | -60.5± 120.5 | -55.2 ± 127.5 | -41.2 ± 99.7 | -53.8 ± 123.2 | 0.53  (0.99) | 0.64 | 0.17 | 0.60 | 0.19 | 0.87 | 0.30** |
| Exacerbation (#/year) | 0.1 ± 0.4 | 0.1 ± 0.6 | 0.02 ± 0.1 | 0.3 ± 0.8 | < 0.001  (< 0.001) | 0.50 | 0.13 | < 0.01 | 0.006 | < 0.01 | < 0.01** |
| Emphysema = % of voxels with CT attenuation <-950 Hounsfield Units (HU) on full inspiration. Functional small airways disease = % of voxels with CT attenuation > -950 HU on the inspiratory exam and <-856 HU on the expiratory scan, as determined via dynamic image registration (Parametric Response Mapping, PRM). Airway thickening = square root of the wall area for a standardized airway with an internal perimeter of 10 mm (Pi10).  * From likelihood ratio test comparing means of 3 groups from multivariable model with outcomes (rows) and group status as predictors adjusted for age, sex, race, smoking history (pack-years) and current smoking.  ^**^ p-values from 2 sample t-test  ^†^ Pairwise p-value form Wald test comparing means of 2 groups | | | | | | | | | | | |

**Table S3**

Comparison of CT variables between ever-smokers with normal FEV_1_ and FEV_1_/FVC > LLN but < 0.70 (“discordant” group, Group 1), ever-smokers with normal FEV_1_ and FEV_1_/FVC > 0.70 (Group 2), never-smokers with normal FEV_1_ and FEV_1_/FVC > 0.70 (Group 3) and ever-smokers with FEV_1_/FVC ≤ LLN and > 75^th^ quartile (Group 4).

|  | | | | | | | | | | | |  |
| --- | --- | --- | --- | --- | --- | --- | --- | --- | --- | --- | --- | --- |
| Variable | **Group 1**  Ever-smokers, Normal FEV_1_,  $\frac{FEV1}{\mathrm{FVC}}<0.7$ and > LLN  (**Discordant Group**)  (n = 161) | **Group 2**  Ever-smokers, Normal FEV_1_,  $\frac{FEV1}{\mathrm{FVC}}$> 0.7  (n = 940) | **Group 3**  Never-smokers, Normal FEV_1_,  $\frac{FEV1}{\mathrm{FVC}}$> 0.7  (n = 190) | **Group 4**  Ever-smokers,  75% quartile < $\frac{FEV1}{\mathrm{FVC}}\leq\mathrm{LLN}$  (n = 379) | Overall p-  value*  Unadjusted  (Adjusted) | P-values for pairwise comparisons (Unadjusted) | | | | | | |
|  |  |  |  |  |  | Group 1 vs. 2 | Group 1 vs. 3 | Group 1 vs. 4 | Group 2 vs. 3 | Group 2 vs. 4 | Group 3 vs. 4 | |
| Emphysema (%) | 2.1 ± 2.9 | 0.7 ± 2.6 | 0.3 ± 0.9 | 2.3 ± 4.4 | < 0.001  (< 0.001) | < 0.001 | < 0.001 | 0.55 | < 0.001 | < 0.001 | < 0.001** | |
| Functional small airways disease (%) | 18.0 ± 10.6 | 9.1 ± 10.0 | 7.1 ± 8.3 | 16.6 ± 11.2 | < 0.001  (< 0.001) | < 0.001 | < 0.001 | 0.16 | < 0.001 | < 0.001 | < 0.001** | |
| Airway wall thickening (Pi10) | 3.70 ± 0.01 | 3.71 ± 0.00 | 3.69 ± 0.01 | 3.73 ± 0.11 | < 0.001  (< 0.001) | 0.41 | 0.01 | 0.008 | < 0.001 | 0.003 | < 0.001** | |
| Emphysema present > ULN | 38.7% | 17.4% | 8.2% | 44.5% | < 0.001  (<0.001) | <0.001 | <0.001 | 0.22 | 0.004 | <0.001 | <0.001^†^ | |
| CT-defined functional small airway abnormality (fSAD) present > ULN | 15.3% | 7.8% | 2.9% | 24.9% | < 0.001  (<0.001) | 0.003 | <0.001 | 0.02 | 0.03 | <0.001 | <0.001^†^ | |
| Either emphysema or fSAD present | 44% | 20.7% | 9.4% | 51.4% | < 0.001  (<0.001) | <0.001 | <0.001 | 0.13 | <0.001 | <0.001 | <0.001^†^ | |
| Both emphysema and fSAD present | 10% | 4.5% | 1.8% | 18.0% | < 0.001  (<0.001) | 0.007 | 0.005 | 0.02 | 0.11 | <0.001 | <0.001^†^ | |
| Presence of emphysema = ≥ upper limit of normal (ULN); Presence of fSAD = ≥ upper limit of normal (ULN). Emphysema = % of voxels with CT attenuation <-950 Hounsfield Units (HU) on full inspiration. Functional small airways disease = % of voxels with CT attenuation > -950 HU on the inspiratory exam and <-856 HU on the expiratory scan, as determined via dynamic image registration (Parametric Response Mapping, PRM).  * From likelihood ratio test comparing means of 3 groups from multivariable model with outcomes (rows) and group status as predictors adjusted for age, sex, race, smoking history (pack-years) and current smoking.  ** P-value from 2 sample t test  ^†^ Pairwise p-value form Wald test comparing means of 2 groups | | | | | | | | | | | | |

Figure S1

Venn diagram illustrating patients within the discordant group (ever-smokers with normal FEV_1_ and FEV_1_/FVC > LLN but < 0.70) who have emphysema, functional small airways disease (fSAD), and both on chest CT imaging.

Figure S2

Density plot of the distribution of emphysema.

Figure S3

Density plot of the distribution of functional small airways disease.
