# Supplementary figures and images for: Heterogeneous burden of lung disease in smokers with borderline airflow obstruction
Source: Respir Res. 2018 Nov 20;19:223. doi: 10.1186/s12931-018-0911-z (PMC6245799; doi:10.1186/s12931-018-0911-z)

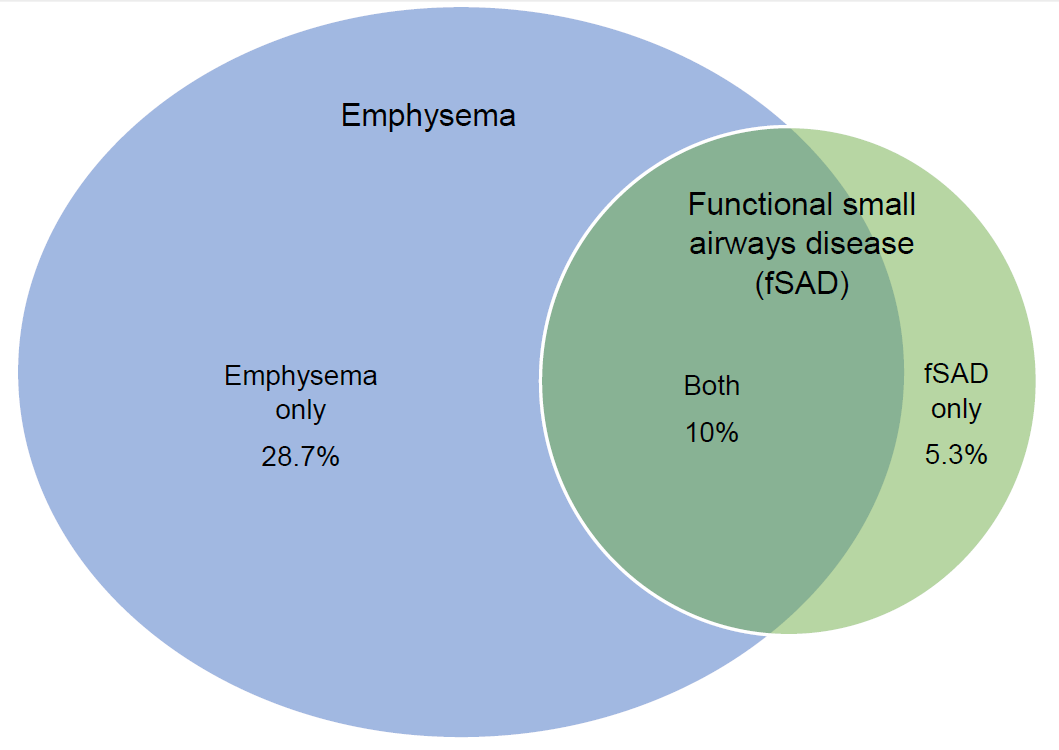

Supplement: Supplementary file 2 — Figure S1. Density plot of the distribution of emphysema. (PNG 58 kb) [file 12931_2018_911_MOESM2_ESM.png]

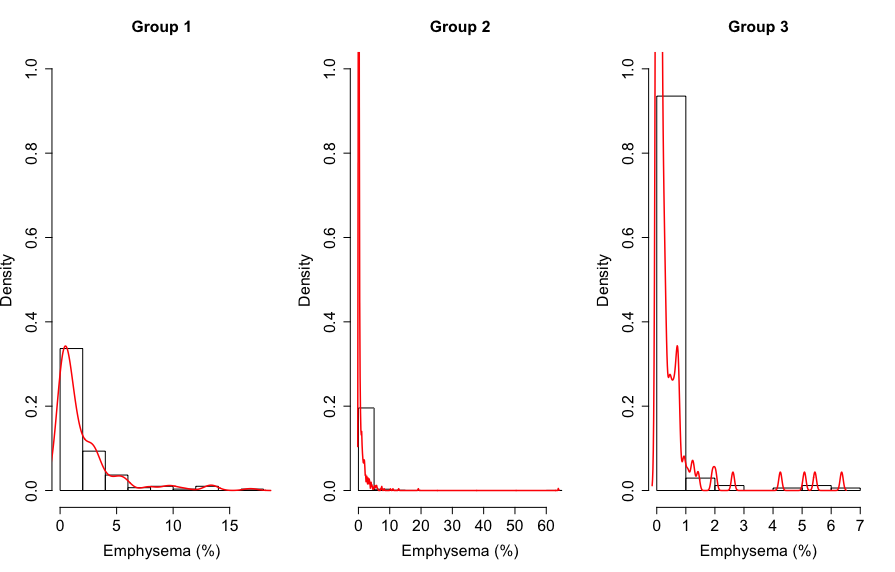

Supplement: Supplementary file 3 — Figure S2. Density plot of the distribution of functional small airways disease. (PNG 55 kb) [file 12931_2018_911_MOESM3_ESM.png]

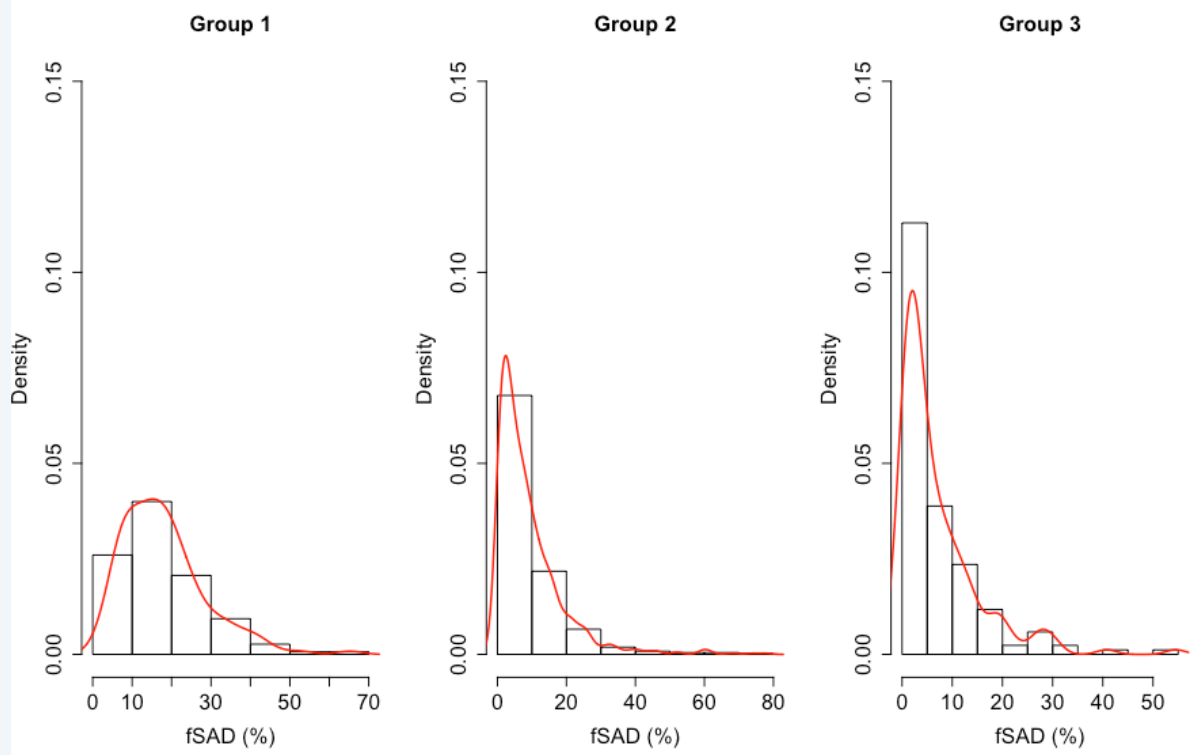

Supplement: Supplementary file 4 — Figure S3. Venn diagram illustrating patients within the discordant group (ever-smokers with normal FEV1 and FEV1/FVC > LLN but < 0.70) who have emphysema, functional small airways disease (fSAD), and both on chest CT imaging. (PDF 34 kb) [file 12931_2018_911_MOESM4_ESM.pdf]
