# Supplementary material for: Heterogeneous burden of lung disease in smokers with borderline airflow obstruction
Source: Respir Res. 2018 Nov 20;19:223. doi: 10.1186/s12931-018-0911-z (PMC6245799; doi:10.1186/s12931-018-0911-z)
Supplement: Supplementary file 5 — Heterogeneous Burden of Lung Disease in Smokers with Borderline Airflow Obstruction. (DOCX 73 kb) [file 12931_2018_911_MOESM5_ESM.docx]

**Heterogeneous Burden of Lung Disease in Smokers with Borderline Airflow Obstruction**

Institutional review board approval reference numbers for each clinical site

| **SPIROMICS Clinical Site IRB Information** | | |
| --- | --- | --- |
| **Clinical Site** | **IRB Review Committee** | **IRB Application Number** |
| CU | Columbia University IRB 2 | IRB-AAAE9315 |
| IA | University of Iowa Hawk IRB-01 | 201308719 |
| JH | Johns Hopkins IRB-5 | NA_00035701 / CR00018131 |
| LA | UCLA Medical IRB 1 (MIRB1) | 10001740 |
| MU | University of Michigan IRBMED B1 Board | HUM00036346 |
| NJ | *Unknown* | 19970 |
| SF | UCSF IRB Parnassus Panel | 10-03169 |
| TE | Temple University IRB A2 | 21416 |
| UA | U of Alabama at Birmingham IRB #2 | 120906004 |
| UI | University of Illinois Institutional Review Board (IRB) #3 | 2013-0939 |
| UT | University of Utah IRB Panel Review Board 5 | 00027298 |
| WF | Wake Forest University IRB #5 | 00012805 |
| *updated 20180315* | | |
